# Lentiviral vector-mediated stable expression of sTNFR-Fc in human macrophage and neuronal cells as a potential therapy for neuroAIDS

**DOI:** 10.1186/1742-2094-8-48

**Published:** 2011-05-14

**Authors:** Shengbo Cao, Chengxiang Wu, Yongbo Yang, Lynn F Sniderhan, Sanjay B Maggirwar, Stephen Dewhurst, Yuanan Lu

**Affiliations:** 1 Department of Public Health Sciences, University of Hawai'i, Honolulu, Hawai'i 96822, USA; 2 Department of Microbiology & Immunology, University of Rochester, Rochester, NY 14642, USA; 3 College of Veterinary Medicine, Huazhong Agricultural University, Hubei, Wuhan 430070, China

## Abstract

**Background:**

Human immunodeficiency virus type 1 (HIV-1) infection frequently causes neurologic disease, which is the result of viral replication and activation of macrophages and microglia in the CNS, and subsequent secretion of high levels of neurotoxic products, including tumor necrosis factor-α (TNF-α). We therefore hypothesized that a soluble TNF-α antagonist might have potential utility as a neuroprotective effecter molecule, and conducted proof-of-concept studies to test this hypothesis.

**Methods:**

To develop novel therapeutics for the treatment of neuroAIDS, we constructed and characterized a soluble TNF receptor (sTNFR)-Fc fusion protein with the goal of neutralizing TNF-α, and tested the stability of expression of this gene following delivery by a lentiviral vector.

**Results:**

High-titer lentiviral vectors were prepared, allowing efficient transduction of macrophage/glial and neuronal cell lines, as well as primary rat cerebellar neurons. Efficient, stable secretion of sTNFR-Fc was demonstrated in supernatants from transduced cell lines over 20 passages, using both western blot and ELISA. Biological activity of the secreted sTNFR-Fc was confirmed by TNF-specific *in vitro *protein binding and functional blocking assays. Finally, the secreted protein was shown to protect neuronal cells from TNF-α, HIV-1 Tat-, and gp120-mediated neurotoxicity.

**Conclusions:**

These results demonstrate that lentiviral vector mediated expression of sTNFR-Fc may have potential as a novel therapy for neuroAIDS.

## Background

HIV-1 associated neurocognitive disorders (HAND), which include asymptomatic neurocognitive impairment (ANI), minor neurocognitive impairment (MND), and HIV-associated dementia (HAD), remain among the most common disorders in people infected with HIV, even in an era when potent antiviral therapy is widely deployed [1]. Indeed, a 2010 study published by the CHARTER Group showed that 52% of HIV-infected adults in a large multisite cohort of more than 1,500 subjects exhibited signs of neuropsychological (NP) impairment, despite the fact some 71% of the persons enrolled in the cohort were receiving combination antiretroviral therapy (cART) at the time of the study [2]. ANI was the most common subdiagnosis in persons with HAND, suggesting that cART may alter the presentation/severity of HAND - even if it has not dramatically changed the overall rate of this disease. This is consistent with reports of more pronounced impairment of executive function and memory/learning in the cART era, compared to the pre-CART period [3]. The inability of cART to prevent HAND, and the failure of many anti-HIV-1 drugs to adequately penetrate the blood-brain barrier (BBB) [4,5], therefore suggest a need for new treatments for this disease.

In the brain, only macrophages and microglia are productively infected by HIV-1 and able to serve as a reservoir for production of progeny virus [6,7]. HIV-1 replication within the CNS also results in persistent activation of brain macrophages and microglia, leading to the secretion of proinflammatory cytokines, particularly TNF-α. TNF-α interacts with two distinct types of cell surface receptors, designated TNF receptor types 1 and 2 (TNFR1, p55 and TNFR2, p75) [8]. TNF-α increases the permeability of the blood-brain barrier, allowing HIV-1-infected monocytes to enter the brain [9]; it also mediates direct neurotoxic effects [10-15].

Present evidence has shown that antagonism of TNF-α by expression of sTNFR can ameliorate inflammatory diseases such as rheumatoid arthritis or reduce TNF-α mediated cytopathicity [16-22]. To explore the efficacy of using genetically modified monocyte/macrophage to deliver sTNFR into the central nervous system (CNS) as a novel treatment for neuroAIDS, we constructed and analyzed an HIV-1-based vector that expresses sTNFR-Fc under the transcriptional control of the human cytomegalovirus (CMV) promoter. This vector was shown to transduce human macrophage and neuronal cell lines stably with high efficiency *in vitro*, resulting in the secretion of high levels of the sTNFR-Fc product. Protein production from the vector-transduced cells remained stable for 20 *in vitro *passages, and the transgene product was shown to be biologically effective as expected (i.e., to functionally block TNF-α activity). Finally, the secreted TNFR-Fc protein was shown to be protective to primary neurons that were exposed to the candidate HIV-1 neurotoxins, Tat and gp120. These studies lay the groundwork for future studies of using sTNFR as a novel therapeutic approach for neuroAIDS.

## Methods

### Cell lines and culture

Human embryonic kidney (HEK) 293T cells were maintained in Dulbecco's Modified Eagle's Medium (DMEM) (Sigma-Aldrich, St. Louis, MO) containing 1.0 g/L glucose, 2 mM L-glutamine, 100 IU/mL penicillin (Sigma-Aldrich), 0.1 mg/mL streptomycin (Sigma-Aldrich) and 10% fetal bovine serum (FBS) (HyClone, Logan, UT). A human neuroblastoma cell line (HTB-11; aka SK-N-SH) and a mouse fibroblast cell line (L929) were cultured in Minimum Essential Medium (Eagle) (MEM) (Sigma-Aldrich) supplemented with 2 mM L-glutamine, 1.0 mM sodium pyruvate, 100 IU/mL penicillin (sigma), 0.1 mg/mL streptomycin (Sigma-Aldrich) and 10% FBS. The human embryonic microglial cell line (CHME-5) was cultured in Dulbecco's Modified Eagle's Medium (DMEM) (Mediatech, Inc., Herndon, VA) containing 4.5 g/L glucose, 2 mM L-glutamine, 100 IU/mL penicillin (Sigma-Aldrich), 0.1 mg/mL streptomycin (Sigma-Aldrich) and 10% FBS.

### Primary Neuronal Cultures

Seven-day-old Sprague-Dawley rats were sacrificed following carbon dioxide inhalation (anesthesia to minimize pain and discomfort) and cerebellar brain tissue was harvested in accordance with Animal Welfare Act and NIH guidelines. The methods used have been described previously [20-22]. In brief, cerebellum was collected, washed, and separated into a single-cell suspension using gentle trypsinization, trituration with a polished glass pipette, and filtration through nylon mesh. Following Percoll density gradient centrifugation to remove glia, the neurons were collected and washed twice in sterile medium without serum, then resuspended in DMEM/F12 Medium with 10% horse serum. Cells were then plated on poly-L-lysine (70K-150K MW; Sigma)-coated 100 mm culture dishes at a density of 3 × 10^6 ^cells per dish. One day later, 5-fluorodeoxyuridine (20 mg/ml) and uridine (50 mg/ml) were added to eliminate proliferative cells (astrocytes); the purity of the neuronal population was verified by immunocytochemical staining for microtubule-associated protein-2. Under these conditions, the cultures were >95% homogeneous for neurons. Neurons were cultured for ≤ 7 days at 37°C in a humidified atmosphere containing 5% CO_2_; and suspended in serum-free DMEM/F12, for 24h prior to the treatments.

### Generation of a HIV-1-based lentiviral vector containing an expression cassette for a human soluble TNF-α receptor (sTNFR)-Fc fusion protein

**Figure 1:**
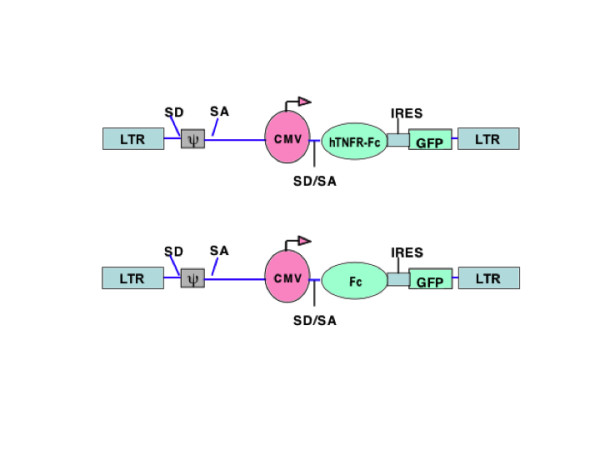
**Lentiviral vector mediated delivery and expression of sTNFR-Fc**. **(A) **Schematic maps of the lentiviral transfer plasmids, pHR-hTNFR-Fc-eGFP and pHR-Fc-eGFP. LTR: long terminal repeat; ψ: packaging signal; SA, SD: splice donor, splice acceptor; CMV: cytomegalovirus promoter; hTNFR-Fc: codon-optimized gene encoding the extracellular domain of the human TNF receptor type 2, fused to the hinge domain from IgG1 and the Fc domain from human IgG3; Fc: hinge domain from IgG1 and the Fc domain from human IgG3; IRES: internal ribosome entry site; GFP: green fluorescent protein.

A transfer plasmid containing an expression cassette for sTNFR-Fc fusion protein (Figure 1) was constructed. Briefly, a human codon-optimized gene encoding the sTNFR-Fc fusion protein was commercially synthesized (GeneArt). This gene contained the extracellular domain of the human TNF receptor type-2 fused through its carboxyl-terminal to the hinge domain from the human IgG1 gene and the Fc domain from the human IgG3 gene. The synthetic gene was then amplified by PCR, using primers containing *Xho *I and *Sac *II restriction sites within the 5' and 3' termini, respectively, and inserted into the pHR-HB7-IRES-GFP plasmid (generously provided by Dr. V. Planelles, University of Utah) that was digested with the same enzymes. The final bicistronic plasmid construct, pHR-hTNFR-Fc-eGFP, expressed the sTNFR-Fc fusion protein and the green fluorescent protein (GFP). A DNA fragment encoding the hinge domain from human IgG1 and the Fc domain from human IgG3 was also amplified similarly through PCR and cloned into the same lentiviral vector plasmid through *Xho *I and *Sac *II digestion and ligation, and used as a control without the TNF-α receptor. Lentiviral vectors encoding the sTNFR-Fc fusion protein, or the Fc fragment alone, were then produced by transient transfection of 293T cells. Vector production, concentration, and titration were performed as described [23-25], except that 293T cells were used for vector titration and initial detection of sTNFR-Fc expression by western blot assay.

### Transduction of human microglial and neuroblastoma cells

Briefly, 5 × 10^5 ^HTB-11 (neuronal) or CHME-5 (microglial) cells were suspended in 0.4 mL RPMI 1640 medium containing 8 μg/mL polybrene in a 1.5 mL tube, and 0.1 mL of vector stock (5 × 10^7 ^IU/mL, MOI = 10) was added and incubated at 37°C for 2 h. Infected cells were then transferred into a 25 cm^2 ^tissue culture flask with 2 mL of fresh growth medium and incubated at 37°C with 5% CO_2_. The medium was replaced 24 h post infection (PI) and transduction efficiencies were evaluated on day 3 PI. The percentage of GFP positive (GFP+) cells was determined by calculating the number of GFP+ and total cells from randomly selected microscopic fields under a fluorescence microscope (Nikon Eclipse TE2000-U). A total of 3 microscopic fields, containing at least 100 cells each, were counted for each transduction test.

### Western blotting

The supernatant or lysate of transduced- or non-transduced- cells including 293T, HTB-11, and CHME-5 cells, was mixed with an equal volume of 2X sodium dodecyl sulfate (SDS) sample buffer (100 mM Tris-HCl at pH 6.8, 200 mM dithiothreitol, 4% SDS, 0.2% bromophenol blue, and 20% glycerol) and loaded on 5% stacking/12% separating SDS-polyacrylamide gels. Following electrophoresis at 30 mA for 1 hr, separated proteins were transferred onto a nitrocellulose membrane (NCM) (Invitrogen, Carlsbad, CA). The membranes were saturated with 1% bovine serum albumin (BSA) (Sigma-Aldrich) in TBST buffer containing 10 mM Tris-HCl at pH 8.0, 150 mM NaCl, and 0.05% Tween-20 for 1 h at room temperature (RT), followed by incubation with diluted goat-anti-human IgG Fc (KPL, Maryland) for 1 h at RT. Following extensive washing with TBST, the NCM was incubated with diluted peroxidase-conjugated rabbit-anti-goat IgG (KPL, Maryland) at RT for 60 minutes, and then washed three more times with TBST and exposed to a 3,3-diaminobenzidine tetrahydrochloride (DAB) substrate (PIERCE, Rockford, IL) for identification of protein bands.

### Enzyme-Linked Immunosorbent Assay (ELISA)

First, a 96-well plate was coated with a goat-anti-human IgG-Fc antibody (KPL) overnight at 4°C. The plate was then washed three times with 0.05% Tween-20 in PBS and blocked with 1% BSA (Sigma-Aldrich) for 30 min at RT on an orbital shaker. After washing three times with PBS, the plate was incubated with diluted sTNFR-Fc containing supernatant samples for 1 h and then incubated with a biotin-conjugated goat-anti-human IgG Fc antibody (Rockland, Gilbertsville, PA) for 1 h. The plate was then washed and finally incubated with streptavidin-horseradish peroxidase (Rockland) for 1 h at RT. The presence of human sTNFR-Fc protein was detected with one-Step Ultra TMB (tetramethylbenzidine) (Pierce). The enzymatic reaction was stopped by addition of 1 M sulfuric acid. The quantitation of sTNFR-Fc protein was based on the optical density values at 450 nm, compared with a standard curve of purified human sTNFR-Fc protein (R&D Systems, Recombinant Human TNF RII/TNFRSF1B/Fc Chimera), using an ELISA reader (Beckman Coulter AD340).

### MTT [3-(4,5-dimethylthiazol-2-yl)-2,5-diphenyltetrazolium bromide] assay

MTT assay [26] was used for cytotoxicity tests. Briefly, test cells at 20,000 cells/well were cultured in 96-well plates at 37°C with 5% CO_2_. After incubation for 24 h, each well was treated with 10 μl MTT (5 mg/mL) for 4 h at 37°C. Cell culture medium was then removed and 100 μl DMSO was added to the wells. Plates were briefly shaken at 60 rpm for 5 min, to dissolve precipitate and remove bubbles, and then read at 570 nm using a microplate reader (Beckman Coulter AD340). The optical densities (OD) of transduced and non-transduced cells at 570 nm were compared and used for evaluating cellular viability.

### Dot-immunobinding assay (DIBA)

Briefly, a nitrocellulose membrane (NCM) strip was equilibrated in TBS and then air-dried. One microgram of standard recombinant human TNF-α protein (PeproTech Inc, New Jersey) was spotted onto a marked area of the NCM and allowed to dry at RT for 30-45 min. The loaded membranes were washed for 5 min in a large volume of TBST and then saturated for 15 min with 3% (w/v) BSA in TBS. The NCM was then incubated for 1 h at RT with cell culture media collected from either transduced or non-transduced control cells, or with recombinant sTNFR-Fc protein (R&D Systems) as a positive control. Following washing with TBST, the membranes were incubated with goat-anti-human IgG Fc-HRP conjugate (KPL) at RT for 1 h. After three washes with TBST, the bound antibody was visualized by incubation with diaminobenzidine (DAB) substrate (Pierce) according to the manufacturer's instruction. Specific binding was visualized by the color deposition on the NCM.

### TNF-α blocking assay

L929 cells at exponential growth phase were harvested and seeded in 96-well plates at a density of 2 × 10^4 ^cells/well and cultured at 37°C with 5% CO_2_. Following a 24-h incubation, cell culture medium was removed and the cells were exposed to the following treatments: 0.1 mL of DMEM containing 8 ng of TNF-α alone; 0.1 mL of DMEM containing a mixture of 8 ng TNF-α and 16 ng commercial recombinant sTNFR-Fc (R&D Systems); or 0.1 mL of a 1:10 diluted conditioned medium collected from either transduced or non-transduced CHME-5 and HTB-11 cells, respectively, containing 8 ng of TNF-α. Triplicate wells were used for each of the treatments including negative controls in which cells were treated with 0.1 mL of DMEM containing no TNF-α. These cultures were incubated at 37°C for 24 h and the relative viability of test cells was then evaluated by performing MTT assay. To examine whether secreted sTNFR-Fc can block TNF-α mediated toxicity on neuronal cells, HTB cells were treated similarly as described for L929 cells. After 3 days of the treatment, cell viability was determined through trypan blue exclusion assay.

### Endogenous TNF-α assay

To rule out the possibility that lentiviral transduction of the macrophage and/or neuronal cells might lead to cellular activation and release of pro-inflammatory mediators such as TNF-α, TNF-α release from the vector-transduced cells was measured by ELISA assay. Briefly, 1.0 × 10^4 ^cells (HTB-11 or CHME-5 transduced with Fc fragment or non-transduced) were seeded into 96-well plates in triplicate wells in 200 μl DMEM containing 2.0% FBS. On day 3 post seeding of the cells, 100 μl of conditioned medium from each well was collected and used to coat 96-well ELISA plates overnight at 4°C. The coated plate was blocked with 1% BSA for 30 min at RT on an orbital shaker. After washing three times with TPBS, the plate was incubated at RT for one hour with 100 μl of 1:10 diluted sTNFR-Fc containing supernatant collected from transduced HTB-11, and the captured sTNFR-Fc was then detected and quantitated with a biotin-conjugated goat-anti-human IgG Fc antibody as described in ELISA assay. This sensitivity cutoff of this method was determined to be 5 pg/mL of TNF-α.

### Neuroprotection assay

To evaluate the ability of the secreted sTNFR-Fc to protect primary rat neuronal cells from TNF-α mediated neurotoxicity, conditioned medium from transduced cells was collected at 48 h of cell growth. Collected medium was diluted 1:10 with MEM containing no FBS, after which it was directly added to primary cultures of neuronal cells that were pre-exposed to HIV-1 Tat protein (500 nM). Control cells received Tat alone. Following a 24-h incubation at 37°C, neuronal apoptosis was measured by TUNEL assays. In addition, the neuroprotective ability of sTNFR-Fc was further verified by treating normal HTB-11 cells (2 × 10^4 ^cells/well) with indicated amounts of Tat or gp120, exposing them to conditioned medium from sTNFR-Fc producing cells, and then measuring cell viability by trypan blue exclusion assays.

### Statistical analysis

Both one-way analysis of variance (ANOVA) followed by a Bonferroni's multiple comparison test and t-test were employed in this study for statistical analysis (using Prism software). * indicates 0.01 < P ≤ 0.05; ** indicates 0.001 < P ≤ 0.01; *** indicates P ≤ 0.001.

## Results

### Characterization of gene transfer efficiency of the sTNFR-Fc expressing lentiviral vector in human neuronal and microglial cells

**Figure 2:**
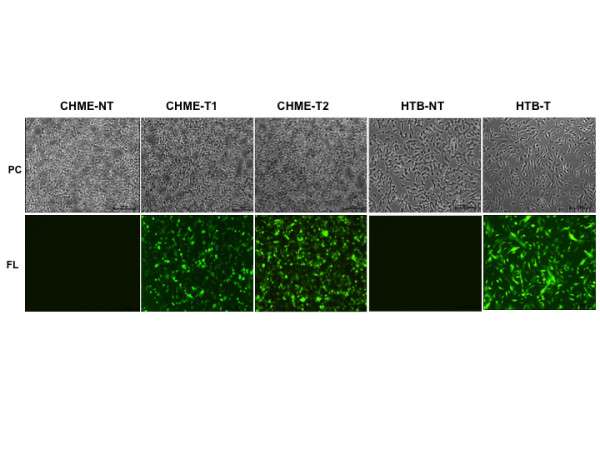
**Photomicrographs of lentiviral vector transudced CHME and HTB cells showing GFP expression**. CHME and HTB cells at exponential growth phase were transduced by the lentiviral vector at an MOI of 10 and transduction efficiency was evaluated by counting the percentage of GFP-positive cells within a transduced cell population using a fluorescence microscope (Nikon Eclipse TE2000-U). Photomicrographs showing transduced cells under phase contrast (PC) bright light or fluorescent light (FL) at 488 nm were taken at magnifications of 100× originally and scale bars in the pictures denote 100 μm. CHME-NT = non-transduced normal CHME cells; CHME-T1 = one transduction and CHME-T2 = two transductions; HTB-NT = non-transduced HTB cells and HTB-T = transduced HTB cells.

Human brain macrophage (CHME-5) and neuroblastoma (HTB-11) cell lines were transduced with lentiviral vectors expressing sTNFR-Fc (Figure 1) at a MOI of 10, and the efficiency of vector-mediated gene transfer was evaluated at day 3 post transduction by counting the number of GFP-positive cells using a fluorescence microscope. The transduction efficiencies were determined to be 65 ± 5% and 100% for CHME-5 and HTB-11 cells, respectively, following a single transduction event (shown as 'CHME-5-T1' and 'HTB-11T'; Figure 2). Gene transfer efficiency in CHME-5 cells was increased to 98 ± 2% following a second transduction with the same vector (CHME-5-T2; Figure 2). In the vector constructs that were used, an enhanced green fluorescent protein (eGFP) was co-expressed through an IRES element to facilitate the monitoring of gene transfer efficiency. Although this approach permitted a convenient assessment of the transfection and transduction efficiencies, it also led to an underestimation of vector-mediated gene expression, since genes expressed through the IRES element are often expressed more weakly than the promoter-proximal gene [27]. To address this concern, sTNFR-Fc expression in CHME-5-T1 cells was further analyzed by conducting indirect immunofluorescence assays using goat anti-human IgG-Fc antibody. Our results revealed that over 80% of the CHME5 cells were sTNFR-Fc positive following a single exposure to the vector (data not shown); this exceeded the estimated gene transfer efficiency, as determined by counting the number of GFP positive cells (65%).

### Stable expression of sTNFR-Fc

**Figure 3:**
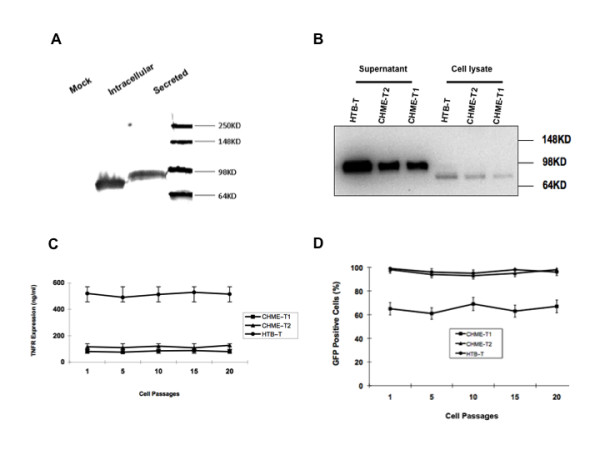
**Stable and high level secretion of sTNFR-Fc in transduced cells**. **(A) **Immunoblot detection of sTNFR-Fc protein in transfected cells. Mock = supernatant from mock transfected 293T cells; Intracellular = transfected cell lysate; and Secreted = supernatant from the transfected 293T cells. **(B) **Immunoblot detection of sTNFR-Fc protein in transduced cells intracellularly (cell lysate) and extracellularly (supernatant). HTB-T = transduced HTB-11 cells; CHME-T2 = CHME-5 cells transduced twice with the vector; CHME-T1 = CHME-5 cells transduced once with the vector. **(C) **Stable expression of sTNFR-Fc protein in conditioned medium of transduced cells. Expression of sTNFR-Fc in supernatants from transduced cells was measured by ELISA; results shown at different cell passages represent mean values from three independent experiments and error bars denote the standard deviation. **(D) **Stable expression of GFP in lentivirus vector-transduced cells. Transduced cells were passed in vitro and the percentage of GFP positive cells was determined every 5 passages by calculating the percentage of GFP+ cells within the culture using an inverted fluorescent microscope (Nikon Eclipse TE2000-U) with a digital camera attachment. Data presented here represent mean values from at least three independent experiments and error bars denote the standard deviation.

Expression and secretion of sTNFR-Fc from the vector construct was first examined by transfection in 293T cells. Robust expression of GFP in transfected cells was readily observed at transfection day 1 (data not shown). To assess sTNFR-Fc protein production extracellularly and intracellularly, culture supernatants and cell lysates from both transfected cells and mock transfected cells were collected/extracted and subjected to western blot analysis. As shown in Figure 3A, there was no detection of sTNFR-Fc expression in the supernatant from mock transfected cells, while vector-transduced cells containing abundant expression of the sTNFR-Fc gene, both within cells (cell lysate) and in secreted form (culture supernatant). The mature, secreted form of sTNFR-Fc migrated more slowly on SDS-PAGE than its intracellular form, with an approximate molecular weight of 95 kD for the secreted form of sTNFR-Fc and 80 kD for the intracellular form of the protein. To confirm the expression of sTNFR-Fc protein from the transduced human neuronal cells, cell-free culture supernatants were collected and subjected to immunoblot analysis. As demonstrated in Figure 3B, sTNFR-Fc protein was readily detected in the supernatant from vector-transduced HTB-11 and CHME-5 cells; the molecular weight of the secreted form of sTNFR-Fc was verified to be approximately 95 kD. When lysates from vector-transduced cells were subjected to the same analysis, an immunoreactive protein of approximately 80 kD was detected. We attribute the apparent difference in the size of the mature, secreted form of the sTNFR-Fc protein relative to its the intracellular form to post-translational modification of the protein during the secretion process (e.g., glycosylation) [28].

To m the level of sTNFR-Fc in culture supernatants, transduced cells were seeded at a density of 1.0 × 10^6 ^cells/mL in a 25 cm^2 ^flask and cultured at 37°C for 24 h. The supernatant was then collected and sTNFR-Fc protein was quantified by ELISA. Results indicated that concentration of the sTNFR-Fc in the supernatant of CHME-5-T1 cells was 80 ± 2 ng/mL; after the second transduction of the cells, the concentration increased to 117 ± 3 ng/mL (Figure 3C). The sTNFR-Fc level in the supernatant from transduced HTB-11 cells was roughly 5-fold higher, at 520 ± 5 ng/mL, following a single transduction. Importantly, sTNFR-Fc expression was not detected in media collected from non-transduced normal CHME-5 and HTB-11 cells under the same conditions (data not shown).

To examine the stability of the sTNFR-Fc expression and secretion, the transduced CHME-5 and HTB-11 cells were serially subcultured 20 times, and sTNFR-Fc levels in the conditioned supernatants were measured by ELISA at every 5^th ^passage, with supernatants collected from corresponding non-transduced cells being used as negative controls. No significant reduction in the sTNFR-Fc expression levels was detected over the course of the 20 passages (Figure 3C). The percentage of GFP positive cells in these transduced cell populations was also evaluated every five passages, and found to be stable over the course of the 20 passages (Figure 3D). These results suggest that the lentiviral vector mediated transduction and sTNFR-Fc secretion was stable and remained at high levels over a prolonged time period.

### Potential adverse impact

**Figure 4:**
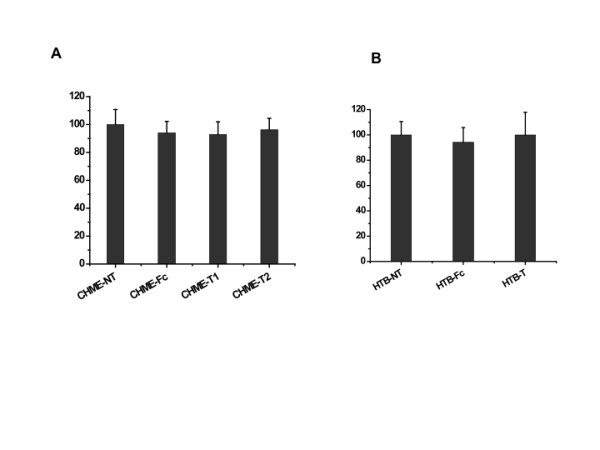
**Comparative analysis of the viability of transduced cells following vector transduction with parental control cells by MTT assay**. The OD570 value for non-transduced cells was arbitrarily defined as 100% cell viability, and data from vector transduced cells was then normalized relative to this. No significant difference was detected among non-transduced (NT), vector-transduced (T), and empty-vector transduced (Fc) CHME (A) and HTB (B) cells (p > 0.05). Results shown represent mean values from three independent experiments and error bars denote the standard deviation.

To determine whether transduction with the lentiviral-TNFR-Fc vector resulted in any adverse impact on target cells, the transduced cells were examined for their growth kinetics and morphology. There were no obvious alterations in cell morphology following transduction, which remained unchanged at different passage numbers (not shown). Subsequent MTT assays also indicated that there was no statistically significant difference in cellular viability (mitochondrial energy metabolism) between transduced and non-transduced control cells (Figure 4).

**Figure 5:**
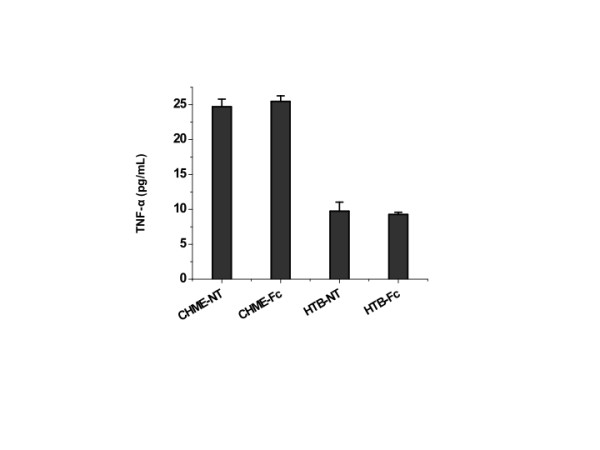
**Transduction with lentivirus vectors does not result in inflammatory activation of CHME-5 or HTB-11 cells**. Endogenous TNF-α secretion into the conditioned media of cells was measured by ELISA. Cells tested were as follows: CHME-NT, HTB-NT: non-transduced CHME-5 or HTB-11 cells; CHME-Fc, HTB-Fc: CHME-5 or HTB-11 cells transduced with the empty vector expressing Fc fragment. Results shown represent mean levels of TNF-α expression from three independent experiments and error bars denote the standard deviation.

We also evaluated whether vector mediated-transduction of CHME-5 or HTB-11 cells resulted in their inflammatory activation. To do this, we measured and compared endogenous TNF-α release by cells transduced with a control lentiviral vector encoding the Fc fragment alone, or non-transduced cells. As shown in Figure 5, endogenous TNF-α release by the vector-transduced macrophage (CHME-5) or neuronal (HTB-11) cells was not significantly higher than that produced by non-transduced cells, indicating that lentiviral transduction did not stimulate the target cells for increased secretion of pro-inflammatory cytokines such as TNF-α.

### Biological function of constitutively expressed sTNFR-Fc

**Figure 6:**
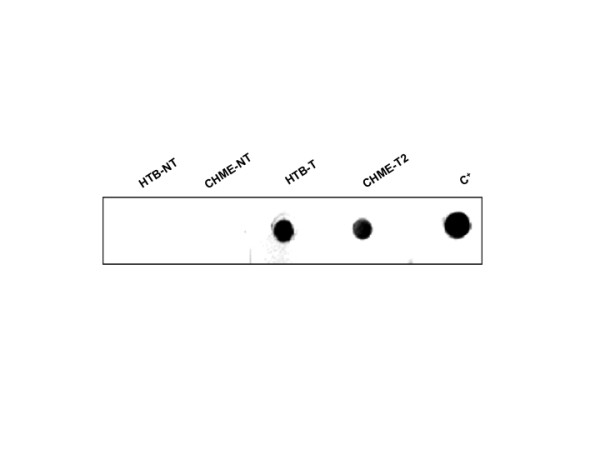
**Specific binding of expressed sTNFR-Fc to TNF-α by dot immunoblot assay**. One microgram of standard recombinant human TNF-α protein was spotted onto nitrocellular membrane (NCM) and then blocked and incubated with supernatants from normal and transduced cells. After three washes with TBST, a goat-anti-human IgG Fc-HRP conjugate was applied and incubated at RT for 1 h. Specific binding was visualized by color deposition on the NCM following incubation with diaminobenzidine (DAB) substrate. Lanes: HTB-NT = supernatant from non-transduced HTB-11 cells and CHME-NT = supernatant from non-transduced CHME-5 cells used as negative controls; HTB-T = supernatant collected from transduced HTB-11 cells and CHME-T2 = supernatant from transduced CHME-5 cells; and C^+ ^= purified recombinant sTNFR-Fc protein as a positive control.

**Figure 7:**
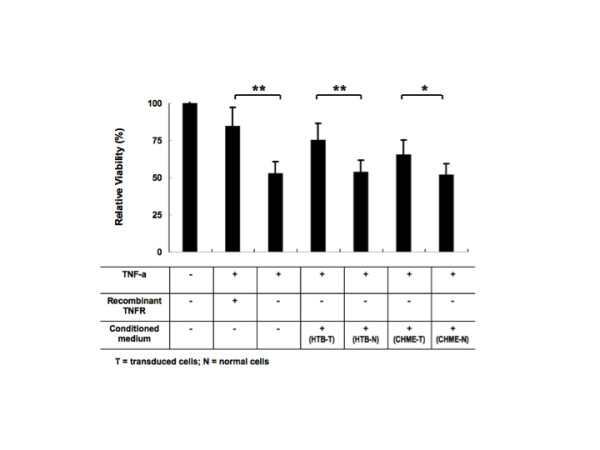
**Functional antagonization of sTNFR-Fc against TNF-α**. As described in materials and methods, TNF-α sensitive L929 cells were treated with TNF-α alone (80 ng/mL) or with TNF-α plus culture supernatants from vector-transduced cells; purified recombinant sTNFR-Fc protein (160 ng/mL) was used as a positive control. After incubation for 24 h, cell viability was then evaluated by MTT assay. Viability was significantly higher for the cells treated with conditioned medium from transduced cells expressing hTNFR-Fc (** p < 0.01 for HTB-T; *p < 0.05 for CHME-T) when compared to cultures that received TNF-α alone, or TNF-α plus culture supernatants from parental cells (CHME-N and HTB-N). Results shown represent mean levels of three independent experiments and error bars denote the standard deviation.

The biological function of the secreted sTNFR-Fc protein was evaluated and confirmed by three different methods. First, a dot immunoblot assay (DIBA) was performed to determine whether the expressed sTNFR-Fc was able to recognize TNF-α. As shown in Figure 6, sTNFR-Fc secreted from both HTB-11 and CHME-5 cells had the ability to bind to TNF-α *in vitro*. Second, the ability of the sTNFR-Fc to antagonize the toxic activity of TNF-α was assessed by using TNF-α-sensitive L929 indicator cells. In this case, an MTT assay was conducted to determine if the secreted sTNFR-Fc protein was able to protect the test L929 cells from the cytotoxic impact of exogenous TNF-α. In this experiment, L929 cells that were exposed to TNF-α in the presence of the culture supernatant (1:10 dilution) from non-transduced control HTB and CHME-5 cells exhibited greatly reduced viability (54+0.7% and 52+0.7%, respectively). In contrast, L929 cells that were exposed to TNF-α in the presence of conditioned media (also diluted 1:10) from vector-transduced HTB and CHME cells were protected from cell killing (viability was 75 + 1.2 and 66 ± 1.4%, respectively) (Figure 7). Control studies confirmed that cells exposed to TNF-α alone underwent high levels of cell death (53 ± 0.9% cell viability), while cells exposed to TNF-α in the presence of 160 ng/mL of commercially available, recombinant sTNFR-Fc (rTNFR) were strongly protected (85 ± 1.4% cell viability). These data indicate that the sTNFR-Fc secreted from vector-transduced cells mediated a significant cytoprotective effect, reflective of its ability to neutralize the biological activity of TNF-α. The purified rTNFR mediated a slightly higher level of cytoprotection (85 ± 1.4% cell viability) compared to 1:10 diluted conditioned medium from vector-transduced HTB and CHME cells (75 + 1.2 and 66 ± 1.4% cell viability, respectively). This reflects the higher concentration of purified rTNFR in this experiment (160 ng/mL), when compared to the level of sTNFR-Fc present in 1:10 diluted cell culture supernatants from the vector-transduced cells (see Figure 3C).

**Figure 8:**
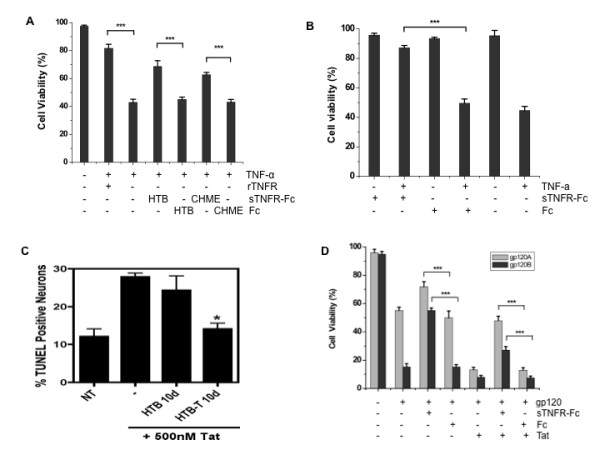
**sTNFR-Fc mediated protection of neuronal cells from TNF-α, HIV-Tat and gp120**. **(A) **Non-transduced human neuronal cells, HTB-11, were treated with TNF-α alone (80 ng/mL), or with TNF-α plus culture supernatants (1:10 dilution) from vector-transduced cells (as indicated). Cells were incubated at 37°C for 3 days, and viability of the cells was determined by the trypan blue exclusion assay. sTNFR-Fc = supernatant collected from HTB-11 or CHME-5 cells transduced with the hTNFR-Fc encoding vector; Fc = supernatant collected from HTB-11 or CHME-5 cells transduced with the vector encoding human Fc only; rTNFR = purified commercial recombinant TNFR-Fc protein (160 ng/mL), used as a positive control. Results shown represent mean levels of three independent experiments and error bars denote the standard deviation. **(B) **Vector transduced human HTB-11 cells were exposed to TNF-α as described in 7A. HTB-11 cells transduced either with the sTNFR-Fc or Fc encoding vector were used in this experiment, along with non-transduced cells as controls. Results shown represent mean levels of three independent experiments and error bars denote the standard deviation. **(C) **sTNFR-Fc protects primary rat neurons against HIV-1 Tat-mediated toxicity. Medium from vector-transduced or parental HTB-11 cells was collected at day 10, diluted and mixed with 500 nM HIV-1 Tat protein, prior to addition to primary rat neurons. Following 24 hours incubation at 37°C, these test cultures along with the control were analyzed by TUNEL assay. Cell death was significantly reduced in neuronal cultures that were treated with Tat plus conditioned medium from HTB-11 cells transduced with the sTNRF-Fc encoding lentiviral vector (HTB-T 10d) versus cells treated with Tat plus conditioned medium from parental HTB-11 cells (HTB 10d) (*p < 0.01). NT, normal neuronal cells received no Tat, and -, neuronal cells exposed to Tat as a positive control. Results shown represent mean levels of three independent experiments and error bars denote the standard deviation. **(D) **sTNFR-Fc mediated neuronal protection of human HTB-11 cells against HIV-1 gp120 toxicity. Conditioned media from hTNFR-Fc or Fc vector transduced HTB-11 cells were collected, diluted and mixed with 100 ng/mL (gp120A) or 250 ng/mL (gp120B) HIV-1 gp120 protein, with or without HIV-1 Tat, prior to addition to HTB-11 cells. Following 3 days incubation at 37°C, cell viability of these cultures, together with control cultures, was determined by trypan blue exclusion assay (***p < 0.001). Results shown represent mean levels of three independent experiments and error bars denote the standard deviation.

As expected, conditioned medium from the control lentiviral-Fc vector transduced cultures had no protective effect on the L929 cells exposed to TNF-α (data not shown). Similarly, sTNFR-Fc expressed from the transduced macrophages (CHME-5) and neuronal cells (HTB-11) was able to protect normal HTB-11 cells from TNF-α mediated toxicity (Figure 8A) and transduced HTB-11 cells expressing sTNFR-Fc were also protected from TNF-α mediated toxicity (Figure 8B).

To evaluate the ability of the secreted sTNFR-Fc protein to block HIV-1 Tat-mediated neurotoxicity, primary rat neurons were treated with recombinant HIV-1 Tat protein in the presence or absence of conditioned medium from vector-transduced cells, and cellular apoptosis was then measured 24 hours later by TUNEL assay. For this experiment, only culture supernatants from transduced HTB-11 cells were tested, since they contain roughly 5-fold higher levels of sTNFR-Fc expression as compared to supernatants from transduced CHME-5 cells (Figure 3C). Neurons exposed to either Tat alone or Tat plus conditioned medium from non-transduced control HTB-11 cells exhibited high levels of apoptosis, as reflected by TUNEL-positive cells (30.7%) while cells exposed to Tat plus conditioned medium from sTNFR-Fc vector-transduced HTB-11 cells exhibited a greatly reduced level of apoptosis (14.2%) (Figure 8C). Since HIV-1 Tat induces neuronal death via production of TNF-α [29-31], our results show that the secreted sTNFR-Fc protein was able to significantly reduce HIV-1 Tat-mediated neurotoxicity by neutralizing TNF-α.

Next, the possibility of blocking the neurotoxicity mediated by HIV-1 gp120, or the synergistic neurotoxicity by gp120 and Tat, was evaluated. As shown in Figure 8D, when HTB-11 cells were treated with 100 ng/mL gp120 for three days, cell viability in cultures co-treated with conditioned media from the sTNFR-Fc vector transduced cells was significantly higher than that in cultures co-treated with conditioned media from the Fc vector transduced cells, or in cultures co-treated with conditioned media from the non- transduced cells (71.9% ± 3.59% VS 49.9% ± 4.76% and 55.2% ± 2.26% respectively). The secreted sTNFR-Fc protein also protected HTB-11 target cells from the combined treatment of HIV-1 gp120 and Tat. In cultures treated with 100 ng/mL gp120 plus 250 ng/mL Tat, cell viability was dramatically increased in cultures that were co-treated with conditioned media from sTNFR-Fc vector transduced cells (47.9% ± 3.17%), compared to cultures co-treated with conditioned media from the Fc vector transduced or non- transduced cells (13.0% ± 1.66% and 13.2% ± 1.78%, respectively). These results were confirmed when a higher concentration of gp120 was used (250 ng/mL) in similar experiments (Figure 8D).

## Discussion

This study employed a lentiviral vector to deliver and express a soluble TNF receptor decoy in microglial and neuronal cells, as a means of protecting cells from TNF-mediated cytotoxicity. The results indicated that both microglial and neuronal cells could be efficiently transduced with the sTNFR-Fc expressing vectors, and the transduced cells showed long-term and stable expression and secretion of sTNFR-Fc. With respect to the expression levels of sTNFR-Fc in these two cell types, quantitative analysis of vector-mediated gene transfer revealed that transduction of the neuroblastoma cells was considerably more efficient than that of the microglial cells. Although the transduction efficiency of the microglial cells was increased to 100% after a second round of transduction, the level of sTNFR-Fc secretion from the transduced cells was still much less than that of the transduced neuronal cells. A potential explanation for this difference in protein expression levels is that HTB-11 cells may have a higher integrated vector copy number of the vector than CHME-5 cells. This is consistent with previous observations that neural cells are more readily transduced by HIV-1 based vectors than cells of myeloid lineage such as macrophages [32]. Alternatively, it is possible that there may be an intrinsic difference in the ability of the two cell types to produce and secrete sTNFR-Fc.

Regardless of the overall efficiency of vector-mediated gene transfer, lentiviral mediated transduction of sTNFR-Fc into neural and microglial cells did not result in any measurable decrease in cell viability. Moreover, transgene expression (of both TNFR-Fc and GFP) was stable in transduced cells over 20 *in vitro *passages. Not only was the expression level stable over time, but also the secreted sTNFR-Fc decoy was shown to be consistently biologically active. DIBA analysis demonstrated that the secreted sTNFR-Fc decoy bound directly to TNF-α, and cell based functional assays revealed that sTNFR-Fc was able to efficiently block TNF-α mediated cytotoxic effects in L929 and HTB-11 cells. Finally, the secreted sTNFR-Fc protein produced by vector-transduced cells was able to protect primary rat neurons and cultured human neuronal cells from HIV-1 Tat and gp120 mediated neurotoxicity, as well as the synergistic neurotoxicity mediated by gp120 and Tat. These findings are significant since HIV-1 Tat is a major virus-derived neurotoxin released by infected macrophages and microglia [30,31,33,34], and gp120 exerts synergistic neurotoxicity with Tat [35,36]. The fact that TNF-α is a major contributor to HIV-1 Tat and gp120 mediated neurotoxicity [37-41] likely explains why sTNFR-Fc is neuroprotective in this setting.

## Conclusions

We constructed an HIV-1-based vector that efficiently transduced human neural and microglial cells, resulting in stable expression and secretion of high levels of sTNFR-Fc. The secreted sTNFR-Fc protein antagonized the biological activity of TNF-α. The secreted sTNFR-Fc protein antagonized the biological activity of TNF-α and protected neuronal cells from HIV-1 Tat-mediated neurotoxicity. These data show that lentiviral vector mediated sTNFR-Fc expression may represent an effective neuroprotective strategy in the context of neuroAIDS. Future efforts to develop this approach further will focus on the establishment of effective methods for *ex vivo *transduction of monocytes using the constructed lentiviral vector, and use of gene-modified monocytes to deliver the therapeutic transgene into the CNS, following migration across the BBB. We believe that this approach has significant potential given the overall favorable safety profile associated with non-CNS penetrant TNF-α inhibitors (including sTNFR-Fc) for treatment of rheumatoid arthritis and other conditions.

## Abbreviations used

TNF: tumor necrosis factor; sTNFR-Fc: soluble TNF-α receptor and human Fc fusion protein; CNS: central nervous system; Fc: fragment, crystallizable.

## Competing interests

The authors declare that they have no competing interests.

## Authors' contributions

SC carried out the vector transduction of CHME and HTB cells and sTNFR-Fc detection, as well as the dot-immunobinding assay and TNF-α blocking assay, and wrote the corresponding part of the manuscript; CW participated in designing the lentiviral constructs and plasmid construction, as well as vector production and transduction of target cells, performed the endogenous TNF-α assay as well as the protection of human neuronal cells from Tat and gp120 mediated neurotoxicity through secreted sTNFR-Fc, and wrote corresponding part of the manuscript; YY ligated the pHR-hTNFR-Fc-eGFP plasmid, and helped SC with vector production, transduction of target cells and sTNFR-Fc detection through western blot and ELISA; LFS & SBM performed the primary neuronal cytotoxicity assay and SBM also contributed to manuscript writing; SD participated in the study design, data analysis and manuscript writing; YL conceived the study, and participated in its design, coordination, data analysis and manuscript writing. All authors read and approved the final manuscript.
